# Utility of colposcopy for the screening and management of cervical cancer in Africa: a cross-sectional analysis of providers’ training and practices

**DOI:** 10.1186/s12913-024-11982-1

**Published:** 2024-12-18

**Authors:** Joël Fokom Domgue, Issimouha Dille, Freddy Gnangnon, Sharon Kapambwe, Celine Bouchard, Nomonde Mbatani, Elodie Gauroy, Nathalie Ledaga Ambounda, Robert Yu, Fatoumata Sidibe, Joseph Kamgno, Bangaly Traore, Pierre-Marie Tebeu, Gregory Halle-Ekane, Mohenou Isidore Diomande, Jean-Marie Dangou, Fabrice Lecuru, Isaac Adewole, Marie Plante, Partha Basu, Sanjay Shete

**Affiliations:** 1 Division of Cancer Prevention and Population Sciences, The University of Texas MD Anderson Cancer Center, 1400 Pressler Dr., FCT4.6002, Houston, TX 77030 USA; 2 Department of Public Health, Faculty of Medicine and Biomedical Sciences, University of Yaounde, Yaounde, Cameroon; 3 Department of Gynecology and Obstetrics, Faculty of Medicine and Biomedical Sciences, University of Yaounde, Yaounde, Cameroon; 4 Centre Inter-Etats d’Enseignement Supérieur en Santé Publique d’Afrique Centrale, Brazzaville, Congo; 5 Division of Non communicable Diseases, World Health Organization Regional Office for Africa, P.O. Box: 06, Brazzaville, Republic of the Congo; 6 Division of Surgical Oncology, Faculty of Health Sciences, University of Abomey-Calavi, Cotonou, Benin; 7 Centre Medical Sante Femmes, Quebec, Canada; 8 Gynecologic Oncology Unit, Groote Schuur Hospital, Faculty of Health Sciences, University of Cape Town, Cape Town, South Africa; 9 Institut Universitaire du Cancer de Toulouse, Toulouse, France; 10 Department of Obstetrics and Gynecology, University Hospital Centre of Libreville, Libreville, Gabon; 11 Medical Oncology Unit, CHU du Point G, Faculty of Medicine and Dentistry, University of Bamako, Bamako, Mali; 12 Division of Surgical Oncology, Faculty of Health Sciences and Technics, University Gamal Abdel Nasser of Conakry, Conakry, Guinea; 13 Department of Obstetrics and Gynecology, Faculty of Health Sciences, University of Buea, Buea, Cameroon; 14 Department of Pathology, University Teaching Hospital of Cocody, Abidjan, Côte d’Ivoire; 15 Department of Gynecologic and Breast Surgical Oncology, Institut Curie, Paris, France; 16 Department of Obstetrics and Gynecology, Faculty of Clinical Sciences, College of Medicine, University of Ibadan, Ibadan, Nigeria; 17 Division of Gynecologic Oncology, Department of Gynecology and Obstetrics, University of Laval, Quebec, Canada; 18 Early Detection, Prevention and Infections Branch, International Agency for Research on Cancer/World Health Organization, Lyon, France

**Keywords:** Colposcopy, Cervical cancer, Cervical cancer screening, Early detection, Transformation zone, Visualization, Healthcare providers, Africa, Secondary prevention, Cancer prevention

## Abstract

**Introduction:**

Cervical cancer is a public health issue in Africa with devastating socioeconomic consequences due to the lack of organized screening programs. The success of screening programs depends on the appropriate investigation and management of women who test positive for screening. Colposcopic assessment following positive screening results is a noteworthy issue in Africa. This study aimed to assess the utilization of colposcopy by providers in the region.

**Methods:**

A cross-sectional study was conducted in 2021–2022 among healthcare providers involved in cervical cancer prevention activities in Africa. They were invited to report prior colposcopy training, whether they performed colposcopy and the indications of colposcopy in their practice.

**Results:**

Of the 130 providers from 23 African countries who responded to the survey (mean age [SD]: 39.0 years [9.4]), half were female (65 [50.0%]), and 90.7% reported working in urban areas. Overall, only 12.6% of respondents indicated having received prior training on colposcopy, and 11.7% reported that they were performing colposcopy in their current practice. Among the providers who reported performing colposcopy in their practice, colposcopy was indicated for routine cervical cancer screening in 21.2% of clinicians, to better visualize the transformation zone in 15.2% of respondents, to further assess the vascularization of cervical mucosa in 33.3% of respondents, and to determine the appropriate treatment modality in 12.1% of respondents. Providers who performed colposcopy in their practice reported a median number of 30 (interquartile range: 19-65) colposcopic procedures in the past 6 months.

**Conclusion:**

Providers’ training and practice of colposcopy for cervical cancer screening remain suboptimal in Africa. To increase utilization of colposcopy in the region, further training is needed to improve providers’ knowledge and engagement. With the development of lower-cost and portable colposcopes, efforts to equip cervical cancer prevention programs and facilities with colposcopy should be enhanced to ensure that women can be screened and managed appropriately in the clinical setting and communities.

**Supplementary Information:**

The online version contains supplementary material available at 10.1186/s12913-024-11982-1.

## Introduction

Assessing women presenting with positive cervical screening results and selecting those suitable for immediate therapy usually relies on the colposcopic assessment of the transformation zone to visualize and characterize cervical epithelium and guide biopsies [1]. In the standard practice, colposcopy guidance is required to perform excision of the transformation zone for treatment of high grade cervical precancers. Thus, colposcopy is an essential part of an effective cervical cancer early detection program.

A major challenge to the successful implementation of cervical cancer prevention activities is the lack of colposcopy utilization following positive screening results. Women’s participation in the entire cervical screening process has been assessed in several studies and barriers to access colposcopy services have been reported [2–4]. Various health systems and patient factors influence access to colposcopy in low and middle-income countries (LMICs). System barriers include a limited number of colposcopy services, which are mostly found in tertiary-level facilities, with long waiting times for patients and few opportunities for non-specialist clinicians to develop the required skills [5]. The limited numbers of trained gynecologists, coupled with the high demands on these doctors for emergency and obstetrical and gynecology services, results in lesser time available for diagnostic or non-urgent procedures like colposcopy [6]. 

A colposcope is a binocular telescope used to directly visualize the cervical mucosa under a good light source. Since it was invented in 1925 [7], it has undergone modifications to improve its diagnostic accuracy and make it more suited to settings with poor health infrastructures. Traditionally, the colposcope has been developed as an optical diagnostic instrument designed for specialists in higher-level healthcare facilities and requires a minimum infrastructure (electricity, examination room, etc.) to operate. In recent years, high-resolution images taken with digital cameras have improved the detection of cervical lesions and enabled images to be shared between senior colposcopists and less experienced ones [8]. Digital colposcopy has several advantages (including cost, portability, and ease of use) that make it more adapted to LMICs compared to its optical counterpart or traditional colposcopes.

In LMICs, the need to create more opportunities for cancer care is growing considering the lack of specialist services and other barriers [9]. Primary healthcare professionals (PHPs) in these settings act as frontline providers in delivering preventive services, including cervical cancer prevention. They strengthen the coordination of care and educate patient using culturally adapted interventions [10]. Not only they assist women in the screening procedure, but also, they provide them with post-screening counseling, orientation and management as appropriate.

The contribution of PHPs in addressing cervical cancer has been highlighted in LMICs [10]. In the innovative approach developed by our team, hands-on training [11] is combined with distant learning through the use of a practical and low-cost tele-mentoring tool (the Project ECHO) aimed at sharing best care practices. In this model, PHPs diagnose and manage patients with the assistance of specialists who act as mentors and provide feedback, guidance, and didactical training [10, 12]. Using this approach, PHPs are equipped with the skills, proficiency, and knowledge to manage cervical pre-invasive or early-invasive disease in their practice. This approach can contribute to lessens travel and wait times, expenses, and potential complications stemming from these postponements. With this tele-mentoring model, PHPs retain their duty of care to patients as their competencies and self-confidence build up, which may reduce referral rates and improve patients’ outcomes [10]. 

Despite the logistical and technological advances aimed at making colposcopy more accessible to LMICs, little is known about African providers’ training, knowledge and practice of colposcopy. Thus, the present study aims to describe the utilization of colposcopy by PHPs involved in cervical cancer prevention activities in Africa.

## Methods

### Study population and study design

The study population consisted of African-based clinicians involved in cervical cancer prevention activities from 23 African countries who were enrolled in a distance learning program focusing on cervical cancer and other HPV-related anogenital diseases [12]. In 2022, providers were invited to take an online survey (in English or French) to assess their training, knowledge, and attitudes toward cervical cancer screening and management of pre-invasive lesions, including the use of colposcopy (see supplementary file). The questionnaire was pre-tested and validated before being administered to the target population. The validation of the survey tool was performed in two steps in accordance with health services research guidelines [13]. In the first step, after developing the survey tool, we shared it with 4 experts to get their feedback and remarks regarding content validity. Suggestions from these experts were then incorporated into the survey tool. In a second step, the self-administered survey was pilot-tested with a convenience sample of 20 individuals of varying healthcare professions based in Africa to ensure clarity of questions and ease of administration. Further comments from this set of HCPs were accounted for in the final revision of the questionnaire. Participation was anonymous and voluntary, and refusal to take the survey had no consequence on participation in the distance learning program. A detailed description of the survey design, content, and administration has been published elsewhere [10]. 

## Measures

### Outcome measures

We assessed whether African-based providers involved in cervical cancer prevention activities performed colposcopy in their practice using the following questions (see supplementary file). “Do you currently perform colposcopy in your practice?” If the answer to this question was “yes,” two follow-up questions were asked: “How many times have you performed colposcopy in the last 6 months?“. In those who reported performing colposcopy in their practice, we also asked the question: “For what purpose do you use a colposcope?”, and the possible responses to this question were: “For routine visual screening,” “To better visualize cervical mucosa,” “To visualize the transformation zone,” “To assess vascularization of the cervical mucosa,” “To determine treatment modality in screen-positive women.” Multiple responses were allowed for this question. Prior training on colposcopy was assessed with the following question: “Have you previously had formal training in performing colposcopy?” (Yes/No).

### Additional variables

To better describe the study population, we collected the following socio-demographic variables: age (years), gender (Male/Female), and location according to the United Nation’s classification of African regions (Eastern Africa, Middle Africa, Western Africa, Southern Africa, and Northern Africa), and setting (urban/rural). Providers were also classified according to their educational background into doctors/residents (including family medicine physicians, internists, obstetricians-gynecologists, oncologists, pediatricians, surgeons, pathologists, etc.), and nurses (including midwives).

In addition to these variables, self-reported knowledge about colposcopy was assessed with the following statement: “My knowledge about colposcopy is adequate for my current practice.” Possible responses to this statement included: “Agree,” “Disagree,” “Neither Agree nor Disagree,” and “I don’t know.”

### Statistical analysis

The descriptive statistical analyses, including prevalence, and associated confidence intervals were obtained using the statistical analysis software SAS (v9.4).

### Ethics approval

This research conformed to the principles embodied in the Declaration of Helsinki. All participants provided written informed consent. The study protocol was approved by the University of Texas MD Anderson Cancer Center’s IRB.

## Results

### Characteristics of the study population

One hundred fifty-three healthcare professionals from Africa completed the survey, including 23 who were non-clinicians (lab technicians, program managers, data scientists, etc...). Among the 130 respondents who were clinicians (mean age [SD]: 39.0 years [9.4]), half were female (65 [50.0%]), and 90.7% reported working in urban areas. Participants were from 23 African countries, including 12 providers (9.2%) from Eastern Africa, 58 (44.6%) from Middle Africa, 6 (4.6%) from Northern Africa, 5 (3.8%) from Southern Africa, and 49 (37.7%) from Western Africa (Table 1).

**Table 1:** Characteristics of the study sample

Variables	*n*	%		95%CI
**Age (years)**				
20–29	22	16.9	10.4	23.5
30–39	59	45.4	36.7	54.1
40–49	26	20.0	13.0	27.0
50–69	23	17.7	11.0	24.3
**Gender**				
Female	65	50.0	41.3	58.7
Male	65	50.0	41.3	58.7
**Facility type**				
Primary	28	24.3	16.4	32.3
Secondary	18	15.7	8.9	22.4
Tertiary	46	40.0	30.9	49.1
Other	23	20.0	12.6	27.4
**Setting**				
Urban	97	90.7	85.0	96.3
Rural	10	9.3	3.7	15.0
**HCP type**				
Doctor/Resident	103	79.2	72.2	86.3
Nurse/Midwife	27	20.8	13.7	27.8
**African Region**				
Eastern Africa	12	9.2	4.2	14.3
Middle Africa	58	44.6	36.0	53.3
Northern Africa	6	4.6	1.0	8.3
Southern Africa	5	3.8	0.5	7.2
Western Africa	49	37.7	29.3	46.1

### Training, practice, and indications of colposcopy

We assessed providers’ prior training and practices regarding colposcopy (Table 2). Only 12.6% of respondents indicated having received prior training on colposcopy, and 11.7% reported that they were performing colposcopy in their current practice.

**Table 2:** Training, self-reported knowledge and practice of colposcopy by African providers

Variables	*n*	%	95% CI		
**Have you previously had formal training in performing colposcopy using a colposcope?**					
Yes	14	12.6	6.3	18.9	
No	97	87.4	81.1	93.7	
**Do you currently perform colposcopy with a colposcope in your practice?**					
Yes	13	11.7	5.6	17.8	
No	98	88.3	82.2	94.4	
**My knowledge about colposcopy is adequate for my current practice**					
Agree	46	50.0	39.6	60.4	
Neither agree nor disagree	13	14.1	6.9	21.4	
Disagree	33	35.9	25.9	45.9	

**Fig. 1:**
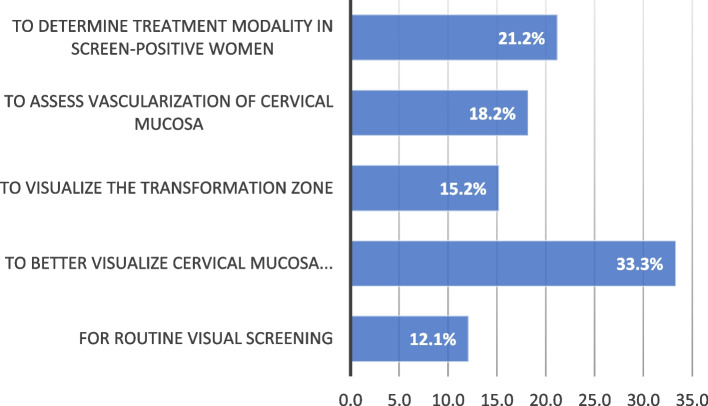
Indications of colposcopy among African clinicians practicing colposcopy

Among providers who reported performing colposcopy in their practice, colposcopy was indicated for routine visual screening in 21.2% of clinicians, to visualize the transformation zone in 15.2% of respondents, to assess vascularization of cervical mucosa in 33.3% of respondents, and to determine treatment modality in 12.1% of respondents. (Fig. 1) In this group, the median number of colposcopies performed in the last 6 months was 30 (Interquartile range: 19–65).

### Perceived knowledge about colposcopy

Providers were also asked if their knowledge about colposcopy was adequate for their current practice. Of the 92 providers who responded to this question, half (50.0%) agreed with this statement (Table 2).

## Discussion

Colposcopy is a critical triaging investigation in the assessment, diagnosis, and management of women with positive cervical screening tests [14]. However, colposcopy (often combined with cervical biopsy) typically is not available in the primary care setting. In many LMICs, the alternative to colposcopy is to visualize the cervix with the naked eye after application of acetic acid and Lugol’s iodine (VIA/VILI), which may result in missed diagnoses [15, 16]. Indeed, the sensitivity of triaging with VIA/VILI in detecting high-grade cervical pre-cancerous lesions among HPV-positive women has been reportedly suboptimal [16, 17]. A key role of colposcopy in the cervical screening continuum is to assess the type of transformation zone and to guid treatment. Studies have reported higher residual disease after excisional treatment (positive excision margin) when treatment was performed without colposcopic guidance [18, 19]. Further, colposcopy evaluation of screen-positive women has proven to reduce the rate of overtreatment, thereby increasing the effectiveness of screening programs [20, 21]. Limited resource settings should be enhanced to provide quality healthcare, particularly when specialized physicians are lacking. Priority interventions include task-shifting and provision of more simple and cost-effective equipment for the diagnosis and management of conditions of public health concern. Our findings that 12.6% of clinicians involved in cervical cancer prevention activities in Africa had been trained at performing colposcopy highlight the lack of skilled colposcopists in the region.

While organizing colposcopy training courses is an effective way to strengthen the diagnostic ability of colposcopists [11], setting-up and evaluating the impact of these courses may be challenging and difficult to scale in practice. Therefore, distant continuing educational programs where expert colposcopists review cervical images of women screened for cervical cancer, can provide a more practical and potentially less expensive opportunity for providers in LMICs to increase their experience and proficiency in accurately performing colposcopy [10, 12]. While harmonized diagnostic standards and quality control procedures for colposcopy practice are released by relevant professional organizations [14], many colposcopists in LMICs, because they lack adequate training, do not rigorously apply colposcopy guidelines in their practice, which leads to unharmonized reporting of colposcopy examinations.

The clinical performance of colposcopy depends on the training and experience of colposcopists and the clinical setting, from basic to referral facilities [22]. When practiced by competent hands, colposcopy is more accurate and may barely miss severe disease, while it can lead to false-positive results when performed by less experienced providers. These weaknesses of colposcopy can be overcome by adequate training, continuing practice, and quality assessment [10, 23]. Although only one in eight clinicians in our study sample had been trained to perform colposcopy, 50% of respondents reported that their knowledge about colposcopy was adequate for their clinical practice. This finding suggests that colposcopy is not considered by many African providers as a procedure that requires specific training and further mentorship, which is a matter of concern as it may have implications on the effectiveness of cervical cancer prevention programs in LMICs.

The use of digital colposcopes as an alternative to optic colposcopes may help improve access to cervical cancer screening and early diagnosis [24]. Previous studies have found that certain digital colposcopes perform comparably to stationary colposcopes [25], including when performed by mid-level providers like nurses [26, 27]. While the feasibility and accuracy of portable colposcopes in LMICs have been reported in demonstration projects, their actual effectiveness in routine clinical practice is yet to be proven, including for the triage of HPV positive women [28, 29]. The relevance of building capacity of HCPs to perform colposcopy in African countries (many of which have a high HIV prevalence) has significantly increased after the publication by the World Health Organization (WHO) in 2021, of the new guidelines for cervical cancer screening and management [30]. This guideline recommended that all women living with HIV and tested positive for HPV require triaging with VIA, cytology, or colposcopy.

In our capacity building model, frontline clinicians (including nurses) are trained to perform VIA/VILI and basic colposcopy [10, 11]. Colposcopy in our study sample was used for routine cervical cancer screening, to better visualize the transformation zone, and to determine treatment modality in screen-positive women. When digital tools used in the context of cervical cancer screening were developed about a decade ago, they were promoted by their manufacturers as devices that can enhance visual screening (especially in settings where primary screening is based on VIA/VILI), and equally promoted as portable colposcopes to distinguish between screen positive women who may require further investigation to guide treatment (especially in settings where primary screening is based on HPV testing). As a result, many providers in Africa utilize these digital and portable tools in different ways depending on their cervical cancer screening strategy, including for primary screening. The practicability of colposcopy performed by trained PHPs using a portable colposcope implies that more women in LMICs can access colposcopy, especially in remote areas.

Considering the dearth of providers skilled to perform colposcopy in Africa, a screening strategy with primary HPV testing followed by optic colposcopy may result in delays in early detection and increase the risk of lost-to-follow up. Therefore, portable colposcopy performed by trained nurses can be offered in the same setting where HPV testing is done and much earlier than a referral to a physician. While colposcopy practice has been limited to medical doctors in most LMICs, our experience shows that PHPs supervised by experienced gynecologists can perform colposcopy with little side effects [10, 11]. In our program, capturing cervical images with colposcopes and other digital equipment helps train PHPs through e-learning sessions (case-based presentation complemented by the review of cervigrams). Moreover, colposcopy with portable devices can be performed during outreaches and generate images that can be used for quality assurance. In the context of Africa where the “see-and-treat” approach (linking cervical cancer screening with treatment of cervical lesions in one or just a few visits) is advocated to reduce the rate of loss to follow up [31], training frontline providers at interpreting cervigrams taken with portable colposcopes on the spot may yield additional benefits in terms of cost-effectiveness and program performance. Not only may it reduce women’s anxiety caused by the wait for their screening results, but it has shown to improve providers’ self-efficacy and self-confidence, as well as coordination of patient care [10]. 

Quality assurance is critical to the effectiveness of a cervical cancer screening program. We employ a collaborative approach for quality assurance through regular meetings in which in-house and external experts discuss cases with attending providers [10]. Adding quality assurance review by specialists using colposcopy has been reported to improve the diagnostic accuracy of VIA [32]. PHPs in more remote facilities can also engage with expert colposcopists through anonymized cervigrams on social media platforms, and colposcopic images can be uploaded to the cloud for quality assurance assessment. This facilitates timely consultation with a distant colposcopist while the patient is still in the healthcare facility and reinforces quality control [33, 34]. 

Artificial intelligence (AI) as an adjunct to colposcopy has raised concerns about the importance of training PHPs in basic colposcopy. Introducing AI-based evaluation and interpretation of cervigrams may reduce the learning curves for PHPs in performing colposcopy. While AI is expected to assist less skilled PHPs in providing more accurate diagnosis [35–37], follow-up of screen-positive women, and treatment of precancerous lesions of the cervix will still require hands-on training and skilled providers.

There were limitations to this study. First, most respondents to the survey were clinicians working in urban settings, suggesting that our findings may not representatively reflect colposcopy practices among providers living in rural areas. Second, respondents were selected among clinicians invited to attend a continuing education e-learning program aimed at building capacity of African providers in the field of cervical cancer prevention and management, and the survey was administered online. As a result, providers in areas with limited internet penetration were underrepresented in our study sample. Furthermore, study participants were healthcare providers involved in cervical cancer prevention activities from 23 African countries who were enrolled in a distance learning program focusing on cervical cancer and other HPV-related anogenital diseases, thus our findings may not be generalizable to all African-based healthcare providers.

## Conclusion

Despite a high incidence of cervical cancer in Africa, provider training, self-reported knowledge, and utilization of colposcopy for cervical cancer screening remain inadequate. To increase access to and use of colposcopy in the region, further training is needed to improve providers’ knowledge and engagement. With the development of lower-cost and portable colposcopes, efforts to equip cervical cancer prevention programs and facilities with colposcopy should be enhanced to ensure that women can be screened and followed up adequately.

## Supplementary Information

Supplementary Material 1.

## Data Availability

The data supporting the findings can be obtained from the corresponding author.
