# Supplementary material for: Utility of colposcopy for the screening and management of cervical cancer in Africa: a cross-sectional analysis of providers’ training and practices
Source: BMC Health Serv Res. 2024 Dec 18;24:1619. doi: 10.1186/s12913-024-11982-1 (PMC11658535; doi:10.1186/s12913-024-11982-1)
Supplement: Supplementary file 1 — Supplementary Material 1. [file 12913_2024_11982_MOESM1_ESM.docx]

**Supplementary File:**

**Questionnaire of African providers involved in cervical cancer prevention and control activities**

**(Questions focusing on colposcopy that were used in the present study)**


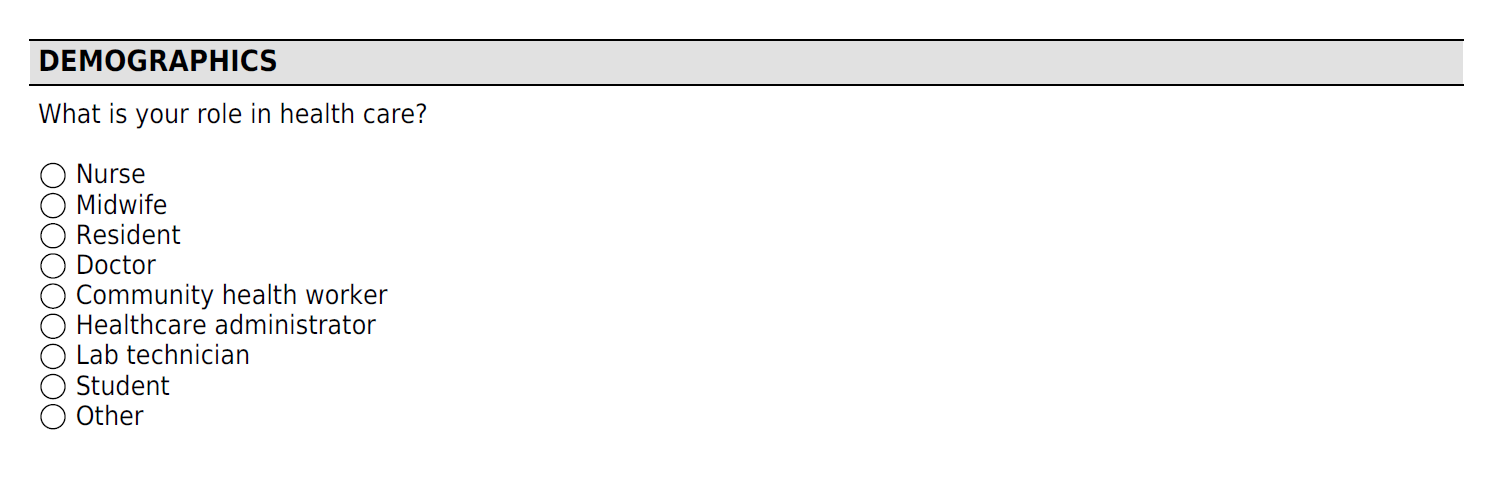

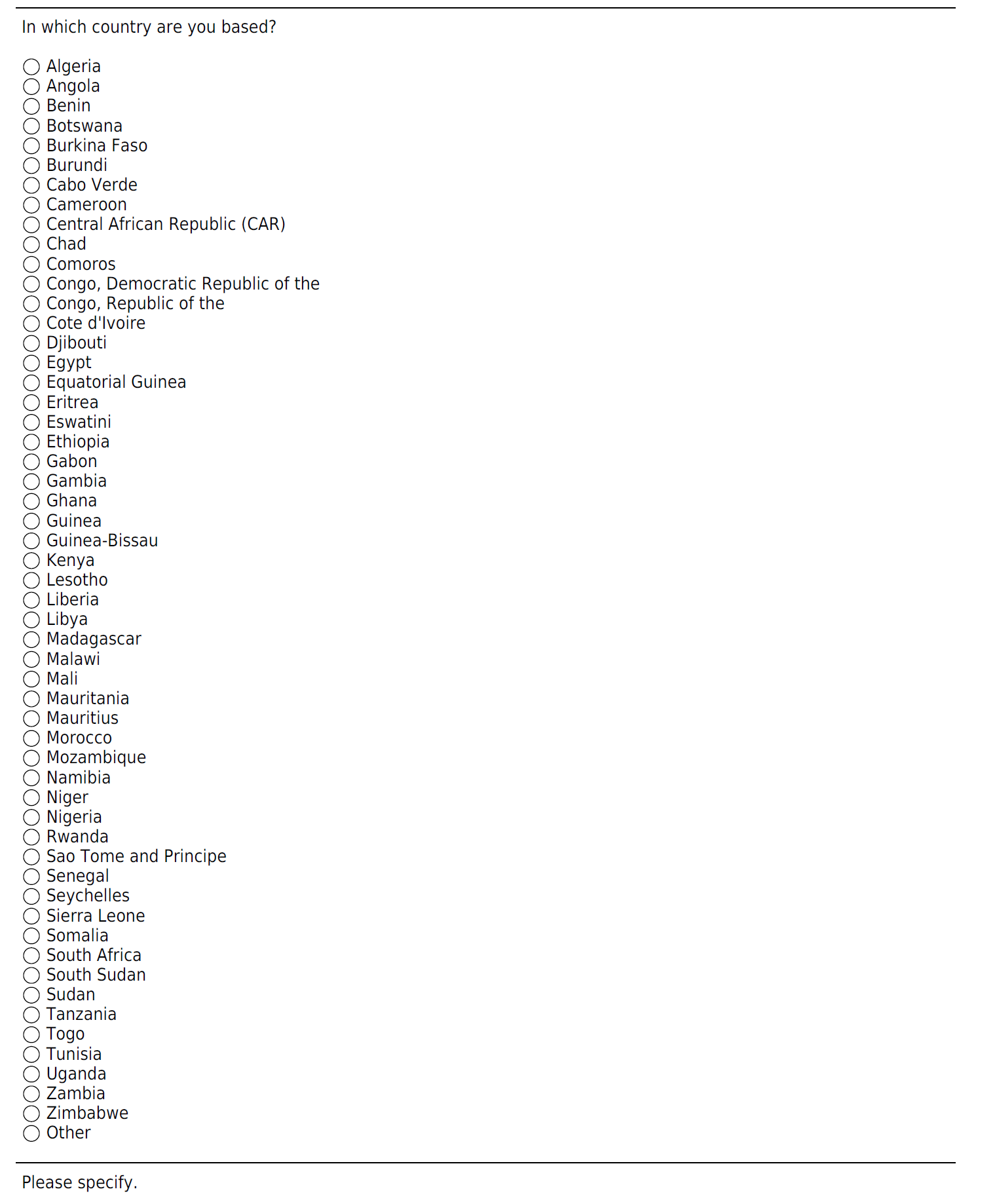


**TRAINING AND COMPETENCIES**___________________________________

Note: The fixed colposcope here refers to the standard colposcope or traditional optical colposcope, while the portable colposcope refers to the hand-held and digital colposcope.

Thank you for your participation in this survey!
